# New somatic mutations and *WNK1-B4GALNT3* gene fusion in papillary thyroid carcinoma

**DOI:** 10.18632/oncotarget.3593

**Published:** 2015-03-14

**Authors:** Valerio Costa, Roberta Esposito, Carmela Ziviello, Romina Sepe, Larissa Valdemarin Bim, Nunzio Antonio Cacciola, Myriam Decaussin-Petrucci, Pierlorenzo Pallante, Alfredo Fusco, Alfredo Ciccodicola

**Affiliations:** ^1^ Institute of Genetics and Biophysics “Adriano Buzzati-Traverso”, CNR, Naples, Italy; ^2^ Istituto per l'Endocrinologia e l'Oncologia Sperimentale (IEOS), Consiglio Nazionale delle Ricerche (CNR), c/o Dipartimento di Medicina Molecolare e Biotecnologie Mediche (DMMBM), Università degli Studi di Napoli “Federico II”, Naples, Italy; ^3^ Department of Pathology, Lyon Sud Hospital Center, Hospices Civils de Lyon, Pierre-Bénite, Lyon, France; ^4^ Instituto Nacional de Câncer - INCA, Praça da Cruz Vermelha, Rio de Janeiro, RJ, Brazil; ^5^ Department of Science and Technology, University “Parthenope” of Naples, Italy

**Keywords:** thyroid, papillary carcinomas, RNA-Sequencing, gene fusions, mutations

## Abstract

Papillary thyroid carcinoma (PTC) is the most frequent thyroid malignant neoplasia. Oncogene activation occurs in more than 70% of the cases. Indeed, about 40% of PTCs harbor mutations in *BRAF* gene, whereas RET rearrangements (RET/PTC oncogenes) are present in about 20% of cases. Finally, RAS mutations and TRK rearrangements account for about 5% each of these malignancies. We used RNA-Sequencing to identify fusion transcripts and mutations in cancer driver genes in a cohort of 18 PTC patients. Furthermore, we used targeted DNA sequencing to validate identified mutations. We extended the screening to 50 PTC patients and 30 healthy individuals. Using this approach we identified new missense mutations in *CBL*, *NOTCH1*, *PIK3R4* and *SMARCA4* genes. We found somatic mutations in *DICER1, MET* and *VHL* genes, previously found mutated in other tumors, but not described in PTC. We identified a new chimeric transcript generated by the fusion of *WNK1* and *B4GALNT3* genes, correlated with *B4GALNT3* overexpression. Our data confirmed PTC genetic heterogeneity, revealing that gene expression correlates more with the mutation pattern than with tumor staging. Overall, this study provides new data about mutational landscape of this neoplasia, suggesting potential pharmacological adjuvant therapies against Notch signaling and chromatin remodeling enzymes.

## INTRODUCTION

Thyroid cancer is the most common endocrine-related cancer, highly heterogeneous in clinical and genetic aspects. Well-differentiated papillary thyroid carcinoma (PTC) constitutes about 85% of all thyroid malignancies [1]. Perturbation of Mitogen-activated Protein Kinase (MAPK) pathway has been frequently described in PTC patients [2]. Mutations in *BRAF* and *RAS* genes (*HRAS*, *KRAS* and *NRAS*) and genomic rearrangements involving *RET* gene (RET/PTC) account for about 70% of PTC cases [3]. In a small percentage of cases (~5%), TRK oncogenes rearrangements have been observed [4]. However, despite the presence of tumor-initiating genetic alterations, cancer results from the progressive accumulation of mutations in genes that confer growth advantage over surrounding cells [5]. Innovative sequencing technologies (Next-Generation Sequencing, NGS, 6) have revolutionized cancer research [7], improving our ability to investigate tumor mutations' landscape. PTC genetic characterization will improve clinicians' ability to establish diagnosis and to predict prognosis and individual response to treatments. Notably, during the writing of the manuscript, a large-scale study exploring single nucleotide variants (SNVs), gene expression and epigenetic features in PTC has been published [8].

Here we describe the identification of a fusion gene and new somatic mutations in PTC patients by RNA-Sequencing. We found, and validated by targeted sequencing, a new chimeric transcript generated by the fusion of WNK lysine deficient protein kinase 1 (*WNK1*) and beta-1,4-N-acetyl-galactosaminyl transferase 3 (*B4GALNT3*) genes. We also discovered new missense mutations in *CBL*, *NOTCH1*, *PIK3R4* and *SMARCA4* genes in PTC patients. Moreover, we report, for the first time in PTC, somatic mutations in cancer driver genes *(DICER1, MET* and *VHL*) described in other tumors. Finally, our analysis confirmed the frequencies of genetic/genomic alterations in *BRAF*, *RET* and *RAS* genes, revealing that these alterations are mutually exclusive and that give rise to distinct gene expression signatures.

## RESULTS

The experimental and computational workflow of our study is schematized in Supplementary Figure 1.

### Detection of known driver PTC mutations

**Figure 1:**
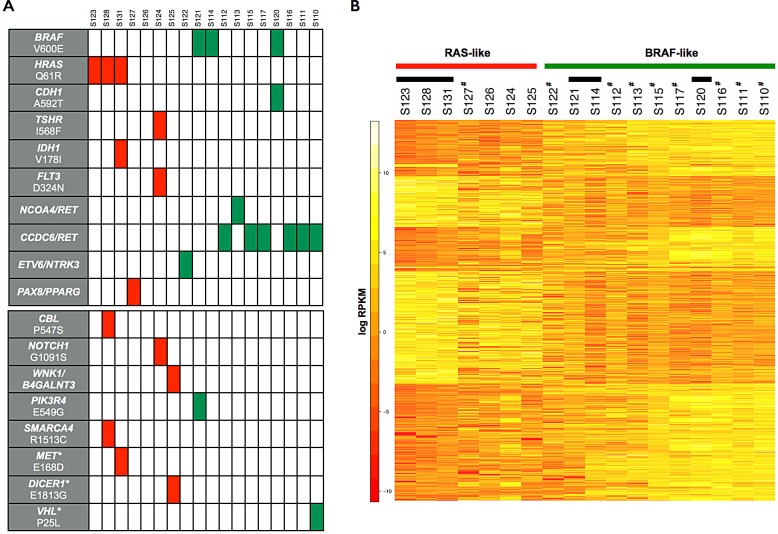
Point mutations, gene fusions and gene expression signatures in papillary thyroid carcinoma A, Schematic representation of protein-altering mutations and gene fusions identified in PTC samples. Each vertical column represents a PTC patient. In the upper panel, known missense mutations and fusion transcripts associated with papillary thyroid carcinoma are shown. In the lower panel are depicted newly identified somatic mutations and other somatic alterations in cancer driver genes reported in other tumors but described for the first time in PTC (indicated by asterisks). Red boxes indicate *HRAS-*mutated patients or those with a RAS-like transcriptional profile. Green boxes indicate *BRAF*-mutated or RET/PTC patients with a BRAF-like transcriptional profile. B, Heatmap of the hierarchical clustering of differentially expressed genes between BRAF-like and RAS-like PTC samples. Black bars indicate samples with point mutations in *HRAS* and *BRAF* genes. ^#^ indicate samples with gene fusions.

To verify if RNA-Seq can reliably identify mutations in driver genes we did not prescreen PTC samples for known mutations/gene fusions. We sequenced paired-end libraries from a discovery cohort of 4 healthy individuals and 18 patients randomly chosen from well-characterized cohort of 80 PTC patients (Supplementary Table 1). As expected by epidemiology and literature data, ~65-70% of tumors had at least one known driver mutation or gene rearrangement. Known mutations identified by RNA-Seq - and validated by targeted sequencing - are summarized in Figure 1A (upper panel). Most of them (~38%) were *RET* gene fusions. Six patients had *CCDC6-RET* (RET/PTC1, ~33%) and one *NCOA4-RET* (RET/PTC3, ~5%) gene fusions, with a PTC1/PTC3 ratio quite similar to that described in literature for patients not exposed to ionizing radiations. RNA-Seq data confirmed *RET* overexpression in these patients (FDR <0.01; Supplementary Figure 2). We also found *PAX8-PPARG* and *ETVN6-NTRK3* gene fusions (~5% frequency each). RNA-Seq data confirmed the overexpression of *PPARG* and *NTRK3* (FDR <0.01; Supplementary Figure 2). Notably, *PAX8-PPARG* chimeric gene, associated to follicular carcinomas, has been reported with low frequency in PTC [8]. RT-PCR and targeted sequencing on cDNAs validated all gene fusions detected by RNA-Sequencing.

Additionally, we found *BRAF*_V600E_ and *HRAS*_Q61R_ (~16% frequency each; Figure 1A), whereas we did not detect *NRAS*/*KRAS* mutations nor TRK gene rearrangements. Notably, *BRAF* and *HRAS* mutations, as well as RET/PTC and other rearrangements, were mutually exclusive in PTC patients. The presence of mutations was confirmed on patients' DNA by targeted sequencing. Such analysis was extended also to negative patients, confirming again the *bona fide* of the SNP calling procedure on RNA-Seq data.

Taking advantage of RNA-Seq data, we correlated global gene expression profiles to known mutations and rearrangements. We found that BRAF-mutated and RET/PTC samples have very similar gene expression patterns and that they differ from RAS-mutated patients (~1400 differentially expressed genes; FDR <0.05). Extending the analysis to PTC patients without any known mutation we found RAS- and BRAF-like gene signatures (Figure 1B). These findings are in agreement with the notion the *BRAF*_V600E_ and RET over-expression activate MAPK pathway more than *HRAS*_Q61R_ and with the recent results of TCGA Consortium [8]. Indeed, we found a significant over-expression - in BRAF- vs RAS-like PTCs - of DUSP genes (*DUSP2*, *DUSP5* and *DUSP6*) that are induced through the stimulation of ERK signaling via MAPK. Conversely, RAS-mutated patients over-expressed anti-apoptotic genes, including *BCL2*.

Finally, we identified known somatic mutations in E-cadherin (*CDH1*_A592T_), in thyroid stimulating hormone receptor (*TSHR*_I568F_), in isocitrate dehydrogenase 1 (*IDH1*_V178I_) and in fms-related tyrosine kinase 3 (*FLT3*_D324N_) genes (Figure 1A). *CDH1* and *IDH1* mutations co-occur with *BRAF* and *HRAS* mutations, respectively (Figure 1A). Mutation frequencies are in line with COSMIC database (~2-5%).

### *CBL, NOTCH1, PIK3R4* and *SMARCA4* genes are mutated in PTC

The reliable identification of known driver mutations using RNA-Seq encouraged us to search for new mutations in cancer-related genes. Combining RNA-Seq data with IntoGen [9] and COSMIC databases [10], we identified - and selected for validation - the following missense mutations (and amino acid changes): c.C1639T (P547S) in *CBL* (proto-oncogene E3 ubiquitin protein ligase), c.G3271A (G1091S) in *NOTCH1*, c.A1646G (E549G) in *PIK3R4* (phosphoinositide-3-kinase regulatory subunit 4 gene) and c.C4537T (R1513C) in *SMARCA4* (SWI/SNF related, matrix associated, actin dependent regulator of chromatin, subfamily a, member 4). Low-frequency mutations in *CBL* and *SMARCA4* co-occur with *HRAS*_Q61R_, *PIK3R4* with *BRAF*_V600E_, whereas *NOTCH1* with *TSHR*_I568F_ and *FLT3*_D324N_ in one patient (Figure 1A). Targeted sequencing on DNAs of positive and negative samples validated the RNA-Seq findings. Mutation frequencies are shown in Supplementary Table 2. Notably, new mutations in *CBL*, *NOTCH1*, *PIK3R4* and *SMARCA4* genes are not annotated as single nucleotide polymorphisms (SNPs) in dbSNP v138 (http://www.ncbi.nlm.nih.gov/SNP/), and in the 1000GenomeProject (http://www.1000genomes.org), nor in COSMIC database. To strengthen these findings, we screened by targeted sequencing 80 alleles from healthy donors and we did not detect any of these mutations.

Noteworthy, RNA-Seq data and targeted sequencing demonstrated that all new mutations are present both on DNA and mRNA, therefore they are not generated by RNA editing. In addition, mutated genes are expressed and it is likely they are translated into mutated proteins. Through *in silico* analyses we attempted to predict the role of these new mutations on protein functionality.

### Possible functional/structural consequences of *CBL, NOTCH1, PIK3R4* and *SMARCA4* mutations

**Figure 2:**
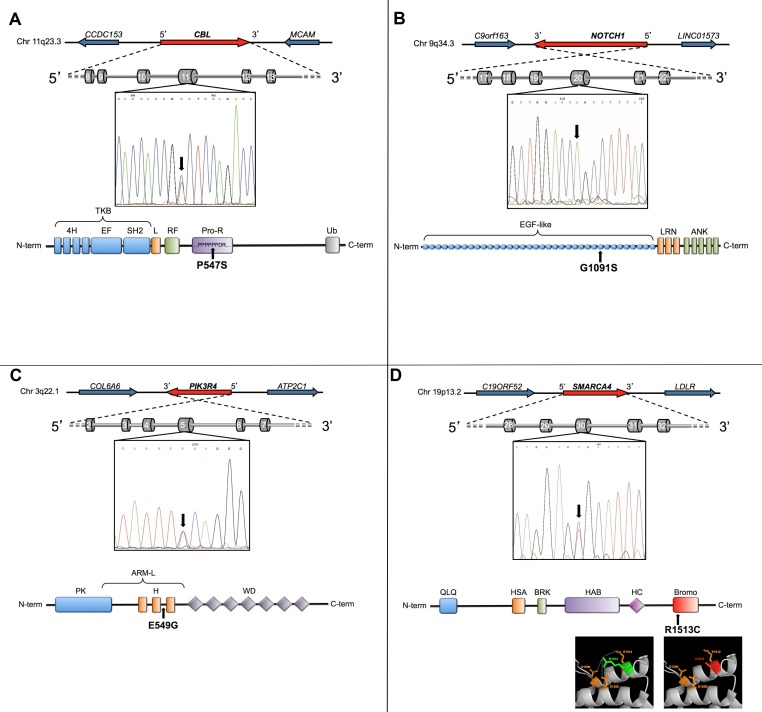
New mutations identified in papillary thyroid carcinoma A-D, the genomic localization and the exon/intron structure of each mutated gene are schematized (A, *CBL*; B, *NOTCH1*; C, *PIK3R4*; D, *SMARCA4*). In each panel, the ectropherogram shows the nucleotide variation identified by RNA-Seq, and the protein graphic representation shows the functional domains affected. In panel D, a detail of the three-dimensional structure of SMARCA4 bromodomain highlights the salt interactions among wild-type residue (colored in green) and the surrounding amino acids (colored in orange). These interactions are lost in the mutated protein (the mutated residue colored in red).

The proline-to-serine amino acid change in E3 ubiquitin protein ligase CBL occurs in a proline stretch (PPPPPPDR) of the Proline-rich domain (Figure 2A). Since the mutated residue is smaller and less hydrophobic than the wild-type it is predicted to affect local folding. Notably, proline residues in this domain are highly conserved and neither the mutant nor other residues with similar biochemical properties have been observed at this position in homologous sequences. Thus, conservation and structural scores indicate the mutation as damaging.

The glycine-to-serine mutation in Notch1 falls in a conserved glycine of a highly-conserved functional region, the EGF-like domain 28 (Figure 2B). Mutant and wild-type amino acids differ in size, charge and hydrophobic properties. Sift and Polyphen scores indicate this mutation as damaging.

The glutamic-acid-to-glycine mutation identified in the phosphoinositide-3-kinase regulatory subunit 4 (PI3KR4) falls in a highly conserved “Armadillo-like helical” domain (Figure 2C). This multi-helical fold, with extensive solvent-accessible surface, is suited to bind large substrates such as proteins and nucleic acids. The mutant and wild-type amino acids differ in electric charge and hydrophobic properties, and the presence of glycine - instead of glutamic acid - is predicted to significantly reduce chain rigidity. *In silico* data indicate that the mutation is potentially damaging to PIK3R4 activity.

The arginine-to-cystein mutation in SMARCA4 occurs in a critical functional region (Figure 2D), the bromodomain (BRD). Structural 3D analysis revealed that the wild-type residue (arginine) forms salt bridges with Asp1506, Glu1512 and Asp1528 and that the mutated residue loses these interactions (details in Figure 2D).

### *WNK1-B4GALNT3*: identification of a novel gene fusion

**Figure 3:**
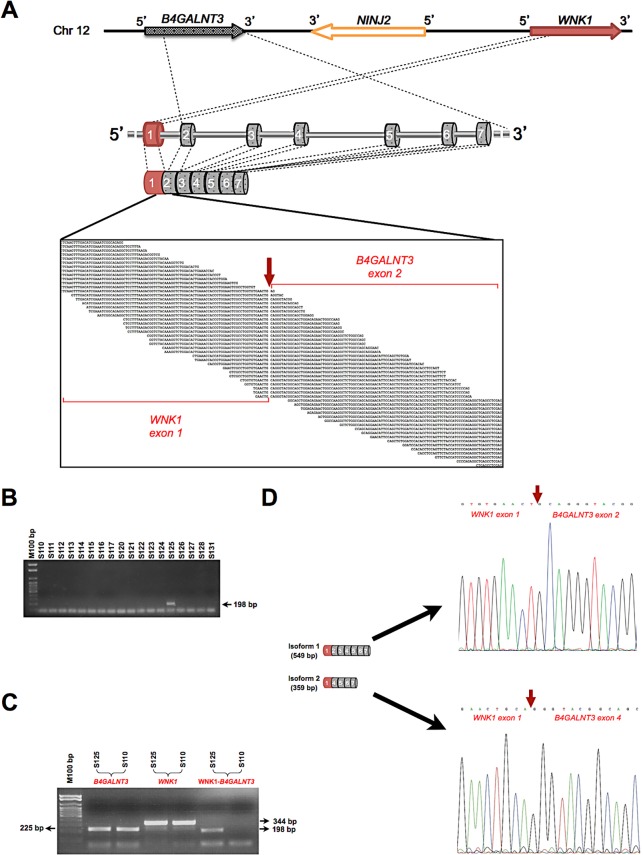
New *WNK1-B4GALNT3* gene fusion in papillary thyroid carcinoma A, Schematic representation of the localization of the fusion partners, *WNK1* and *B4GALNT3,* on chromosome 12. The exons of *WNK1* and *B4GALNT3* genes that are involved in the fusion are indicated in red and grey, respectively. The RNA-Sequencing reads that map across the fusion breakpoint are shown in the black box. The red arrow indicates the exact fusion breakpoint. B, RT-PCR validation of the *WNK1*-*B4GALNT3* fusion performed on the RNA of 18 PTC samples of the discovery cohort. C, Qualitative RT-PCR assay on the mRNAs of *WNK1* and *B4GALNT3* parent genes. Agarose gel picture shows the PTC sample with the fusion and a negative sample. D, Schematic mRNA structure of the two isoforms of *WNK1*-*B4GALNT3* fusion gene. The electropherograms show the nucleotide sequences of the breakpoint (indicated by red arrows).

Computational analysis of chimeric transcripts revealed the presence of known gene fusions (*CCDC6-RET*, *NCOA4-RET*, *PAX8-PPARG* and *ETVN6-NTRK3;* Figure 1A). Moreover, we identified a new *WNK1-B4GALNT3* chimeric transcript in one patient, negative for known PTC-causing genetic alterations. RNA-Seq data indicated that the new chimeric transcript originates by fusion of the exon 1 of *WNK1* and the exon 2 of *B4GALNT3* (Figure 3A). Fusion-specific RT-PCR and sequencing confirmed the fusion breakpoint in the transcript, revealing also that it undergoes alternative splicing (Figure 3B). Notably, sequence and ORF analysis revealed that the longest fusion transcript is out-of-frame whereas the alternative isoform keeps the ORF intact. We demonstrated that this patient does not carry reciprocal gene fusions. In addition, since RNA-Seq indicated that the canonical mRNAs of *WNK1* and *B4GALNT3* genes are transcribed in this patient, we confirmed it by RT-PCR (Figure 3C). Using the same approach, we validated the absence of gene fusions in negative patients (Figure 3D). In line with the over-expression of *RET*, *PPARG* and *NTRK3* in patients with gene rearrangements, RNA-Seq data showed *B4GALNT3* over-expression in this patient (p <0.05; Supplementary Figure 2). The expression of *WNK1* was not affected. Fusion partners map on chromosome 12 (chr12p13.33), are transcribed from the same strand (5′-3′ orientation) and are separated by about 220 Kb (Figure 3A). It indicates that the fusion derives from an intrachromosomal paracentric rearrangement. We could not identify in this patient, or in other patients of the discovery cohort, additional fusions involving genes mapping in the same genomic region.

### Mutations in known cancer driver genes

The filtering procedures during the SNP calling retrieved ~7430 missense, stop gain/loss point mutations and INDELs. We focused on 125 “Mut-driver” genes, defined by Vogelstein and colleagues [5] as those containing a sufficient number of driver mutations to unambiguously distinguish them from other genes. We found 44 variants in 32 genes (Supplementary File 1). Notably, most of the genes that we found mutated for the first time in PTC (i.e. *CBL, NOTCH1, SMARCA4, MET* and *VHL*) are “Mut-driver” genes. Figure 1 shows whether mutations in these genes co-occur with known oncogenic mutations, gene rearrangements and fusions.

**Figure 4:**
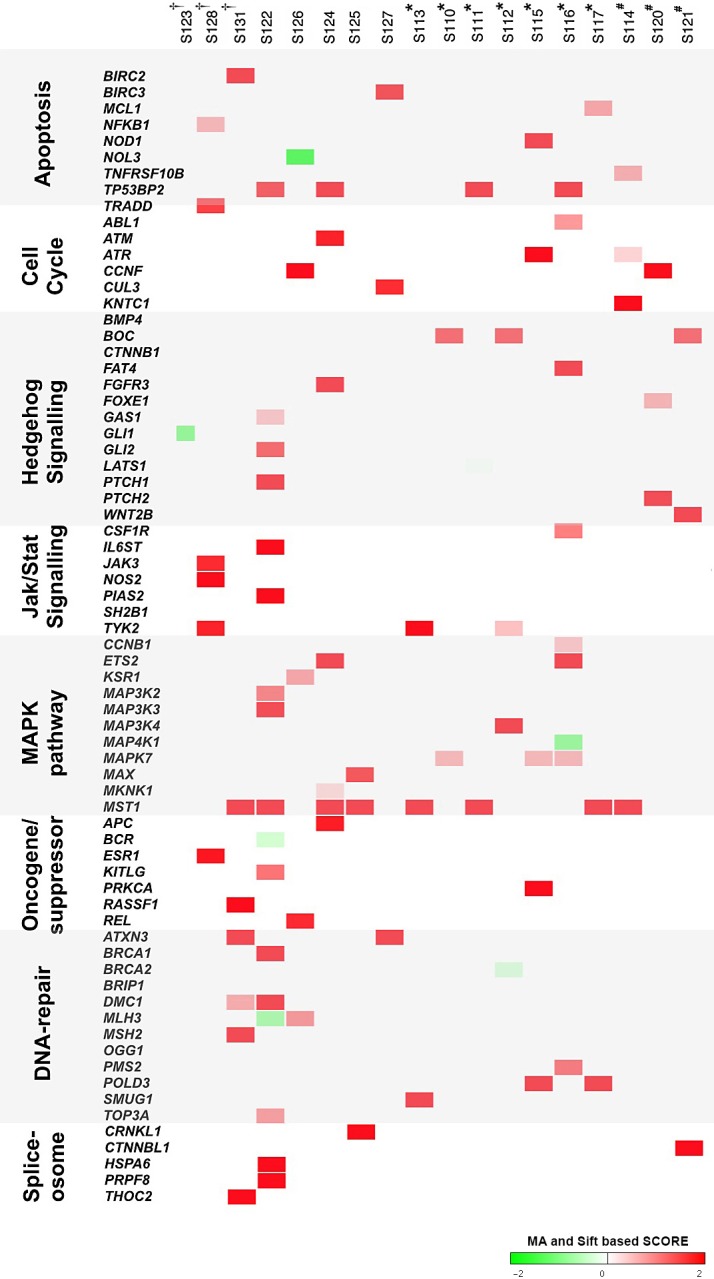
Genomic landscape of PTC mutations and related pathways Co-occurrence of protein-altering nonsense and missense mutations identified in PTC patients (n=18) by RNA-Sequencing. Most relevant shared mutations in biological pathways associated to tumorigenesis are shown. Each vertical column represents a PTC patient. *HRAS*_Q61R_, *BRAF*_V600E_ and RET/PTC patients are indicated by ^†^, ^*^ and ^#^, respectively. The severity of the amino acid change is proportional to the intensity of red and green boxes (according to MA, “Mutation Assessor”, and Sift scores).

Moreover, we investigated known cancer driver genes and their interacting partners in pathways commonly associated to tumorigenesis. We extensively searched damaging mutations affecting JAK-STAT signaling, MAPK, apoptosis, cell cycle, Hedgehog, oncosuppressors and oncogenes, DNA-repair and spliceosome pathways (Figure 4). We found 61 mutated genes in these pathways, some of them annotated as cancer driver in IntoGen [9]. We identified damaging mutations in *ATR*, *BRCA1*/*2*, *MAP4K1*, *CUL3* and *MAX*, already reported in other cancer types but not described yet as mutated in PTC. Although most of them are not classified as cancer drivers, some mutations discovered here in PTC for the first time - *MST1*_R703C_ and *BOC*_Q915H_ - are somatic in COSMIC [10].

## DISCUSSION

Our study describes the identification in PTC of a novel gene fusion, new somatic mutations in established cancer driver genes and known mutations (reported in other cancer types) not described yet in PTC. Taking advantage of next-generation RNA-Seq, we found that BRAF- and RAS-mutated tumors have distinct gene expression profiles, and that RET/PTC samples resemble tumors with BRAF_V600E_ mutation, in line with the very recent results of TCGA [8]. We found that gene expression is mostly correlated to specific genetic alterations rather than tumor stage, suggesting the importance to genetically characterize PTC patients.

We confirmed that driving somatic mutations and rearrangements are mutually exclusive in PTC. Accordingly, the new *WNK1-B4GALNT3* fusion has been discovered in a patient negative for known PTC-inducing alterations. Noteworthy, a significant over-expression of *B4GALNT3* gene was found in this patient, whereas the fusion partner was not affected (Supplementary Figure 2). *B4GALNT3* can act both as tumor suppressor in neuroblastoma [11] and as oncogene in the colon, increasing the malignant phenotype of colon cancer cells through enhanced integrin and MAPK signaling [12]. Its expression positively correlates with metastasis and poor survival in patients with colorectal cancer [13]. Our data suggest a new role of *B4GALNT3* as oncogene in PTC. Notably, *WNK1-B4GALNT3* positive patient also carries a somatic mutation in *DICER1*, cancer driver gene. The mutation affects the metal binding site of RNase IIIb domain, recently found mutated in non-epithelial ovarian cancers [14]. To the best of our knowledge, *DICER1*_E1813G_ mutation has not been previously described in PTC.

Furthermore, we discovered a new missense *NOTCH1*_G1091S_ mutation in a patient negative for *BRAF*/*RAS* mutations and RTKs rearrangements. Many *NOTCH1* driver mutations have been reported in hematopoietic tumors, head and neck squamous cell carcinoma and other malignancies [15]. The presence of inactivating mutations indicates this gene as tumor suppressor, rather than oncogene, in solid tumors [15], [16]. Interestingly, most of the mutations in solid tumors are clustered within EGF-like repeats. Accordingly, the *NOTCH1*_G1091S_ mutation falls in the EGF-like domain 28 and affects a highly conserved residue. Although 3D model revealed this domain does not directly bind Notch ligands, Sharma and colleagues reported it to interact with EGF-like 11-15 domains, crucial for receptor activity [17]. Thus, this mutation may affect Notch1 protein functionality and Notch signaling that is directly linked to PTC cell proliferation [18]. These results suggest that this pathway should be taken into account as adjuvant therapy for treating PTC. The same mutation co-occurs with two low-frequency mutations (*TSHR*_I568F_ and *FLT3*_D324N_) previously reported in PTC.

Additional missense mutations have been discovered in *BRAF*- and *RAS*-mutated patients. Among these, *CBL*_P547S_ affects the proline-rich region, responsible of the binding with SH3 domain of Grb2 that indirectly recruits it to RTKs via GRB2 adaptor protein [19]. In lung cancer, mutations in *CBL* and in other driver genes usually co-occur [19]. Indeed, the new *CBL* mutation was found in a *HRAS*-mutated patient. Interestingly, the same patient carried a new missense mutation in the tumor suppressor gene *SMARCA4*, frequently mutated in lung cancer and small cell ovarian carcinoma [20], [21]. Such mutation disrupts salt interactions with charged residues in the BRD, a functional domain that allows the recognition of acetyl lysine marks on H3 and H4 tails [22]. SMARCA4 protein, associating with Rb proteins, induces cell cycle arrest through HDAC-dependent transcriptional repression. Mutations, rearrangements or over-expression of BRD-containing proteins have been reported in tumors, and BRD inhibitors have been developed to induce cycle arrest and apoptosis of carcinoma cells [22]. Therefore, a similar pharmacological approach could be adopted in the treatment of PTC cases with *SMARCA4* mutations.

Finally, other mutations - confirmed somatic in other cancer types - were identified for the first time in PTC. *MET*_E168D_ in the SEMA domain, crucial for the interaction with plexins, has been previously described in small cell lung cancer. Interestingly, this mutation impairs the affinity for HGF and alters MET functionality [23]. We also found nucleotide variations in *ATM* gene. This finding is relevant since PTC is the most frequent radiation-sensitive tumor, and ATM is a fundamental kinase that triggers the DNA damage checkpoint, determining cell cycle arrest, DNA repair or apoptosis.

In this study we have confirmed that next generation RNA-Sequencing is a powerful approach to simultaneously identify gene expression profiles, gene fusions and mutations in cancer samples. One of the major limitations in this kind of approach is that only mutations in expressed genes can reliably be detected. However, although it may limit the SNP calling in RNA-Seq data, it is proven that most of mutations responsible of tumor phenotypes fall in the protein coding regions of actively transcribed genes and lead to mutated proteins. In conclusion, our data confirm the genetic heterogeneity of PTC and reveal the accumulation of mutations in new driver candidate genes. These findings pave the way to novel potential pharmacological therapies in PTC treatment, based on the presence of new affected pathways, such as Notch signaling and chromatin remodeling.

## MATERIALS AND METHODS

### Patients and RNA samples preparation

Thyroid biopsies of 18 PTC and 4 control thyroids constituted the discovery cohort for RNA-Sequencing. Tumor specimens were not prior genetically characterized. Biopsies from other 32 PTC patients and 26 healthy thyroids constituted the validation cohort. All biopsies were obtained from the Service d'Anatomo-Pathologie, Centre Hospitalier Lyon Sud, France. Informed written consent was obtained from patients of both cohorts. Approval of the ethic committee of University of Naples “Federico II” and Lyon Sud Hospital Center was obtained to use these samples for RNA-sequencing, Sanger sequencing and validation purposes. Total RNA was extracted from biopsies using Trizol standard procedure and RNA integrity was assessed using digital gel electrophoresis (Experion) and spectrophotometry as previously described [24].

### Library preparation and RNA-Sequencing data analysis

Paired-end libraries were prepared using TruSeq RNA Sample Preparation Kit (Illumina) and sequenced on Illumina HiSeq2000 platform according to manufacturer's instructions. Details of RNA-Seq data analysis are given in Supplementary Methods Section. Briefly, reads mapping was performed using TopHat v.2.0.7 [25], PCR duplicates were removed using Picard tools v1.117 (http://broadinstitute.github.io/picard/) and SNP calling was carried out using GATK v3.3 workflow optimized for RNA-Seq reads [26]. Nucleotide variants were annotated using ANNOVAR [27] and COSMIC database [10] was used to label variants as “somatic in cancer”. Common germ-line variants annotated in dbSNP v138, 1000 genomes as well as those identified in healthy thyroids were filtered out. SNVs in super-duplicated regions were also removed. For gene expression analysis, read counts per gene was obtained with HTSeq [28] and RPKM (Reads per kilobase transcript per million reads) normalization was used [32]. Fusion transcripts were identified using TopHat Fusion [30] and Chimerascan v0.4.5 [31].

### Targeted DNA sequencing and mutation detection

Genomic DNA was isolated from 80 thyroid biopsies (50 PTC and 30 healthy thyroids) and 40 peripheral blood samples (healthy donors) using standard lysis/phenol extraction protocols as previously described [32]. Known and newly identified point mutations (Supplementary Table 2) were validated in tumor DNA using gene-specific PCR assays and Sanger sequencing. New mutations in *CBL*, *NOTCH1*, *PIK3R4* and *SMARCA4* genes were screened on genomic DNA of healthy individuals. Oligonucleotides sequences are provided in Supplementary Table 3.

## SUPPLEMENTARY METHODS, FIGURES AND TABLES


