# Supplementary material for: New somatic mutations and WNK1-B4GALNT3 gene fusion in papillary thyroid carcinoma
Source: Oncotarget. 2015 Mar 14;6(13):11242–51. doi: 10.18632/oncotarget.3593 (PMC4484453; doi:10.18632/oncotarget.3593)
Supplement: Supplementary file 1 [file oncotarget-06-11242-s001.pdf]

New somatic mutations and *WNK1-B4GALNT3* gene fusion in papillary thyroid carcinoma

Supplementary Methods

Sequence analysis

RNA-Seq was performed at high coverage on a total of 18 papillary thyroid cancer tissues and 4 normal thyroids. A total of about 2,2 billion of paired-end reads (75+75 and 100+100), corresponding to ~180Gb (gigabases) were sequenced. Reads' quality was assessed using FastQC (http://www.bioinformatics.babraham.ac.uk/projects/fastqc/) softaware. TopHat2 version 2.0.10 [1] was used to map RNA-Seq reads against a known transcriptome annotation (Ensembl v70) – in a first step – and then against the reference human genome (hg19). The transcriptome-mapping step improves the overall sensitivity and accuracy of reads mapping. Parameters used for the mapping with TopHat2 were: -p 12 -N 2 -g 10 -r 200 -a 15 -m 1 -i 100 --library-type fr-unstranded --fusion-search --segment-mismatches 3 --read-edit-dist 2 --transcriptome-index.

Ensembl v70 track was downloaded from UCSC Table Browser (http://genome.ucsc.edu). Only uniquely mapped reads (about 95% of sequenced reads, Supplementary table1) were used for further analyses. Coverage files were produced using BEDTools v2.17.0. Visual inspection of reads and coverage files on UCSC Genome Browsers was used to assess the overall quality of the RNA-Seq experiment, and to inspect gene-specific features of interest. Gene expression quantification was performed using HT-Seq [2]. Normalized gene expression values (RPKM) were used to identify differentially expressed genes using complex design implemented in EdgeR algorithm to remove batch effects. Gene ontology and pathway analysis were performed using DAVID [3].

Analysis of fusion transcripts

Fusion transcripts discovery was performed using the algorithms TopHat Fusion (4; parameters: --num-fusion-reads 5 --num-fusion-pairs 4) and Chimerascan (5; default parameters). Fusion transcripts with less than 7 spanning reads (reads that map across the fusion breakpoint) were filtered out. Moreover, we removed fusion events observed in adjacent and/or overlapping genes as well as fusions involving HLA, IGH genes and other involving genes from repeated families.

Analysis of single nucleotide variants

PCR duplicates reads were removed by using Picard tools v1.93 (http://broadinstitute.github.io/picard/). Subsequently, we used GATK best practices recommendations for calling variants on RNA-Seq data. These recommendations are based on classic DNA-focused Best Practices, with key differences in the early data processing steps (focus on handling splice junctions correctly), as well as in the calling step [6]. Details about he GATK best practices for variant calling on RNA-Seq are provided in GATK forum (http://gatkforums.broadinstitute.org/discussion/3892/the-gatk-best-practices-for-variant-calling-on-rnaseq-in-full-detail).

In order to filter the resulting callset, variants with clusters of at least 3 SNPs that were within a window of 35 bases and variants with a Quality By Depth values (QD < 2.0) were filtered out. Moreover, the variants with a recalibrated score < 30 and the predictions supported exclusively by variants located in the beginning or the end of the reads were filtered out. The final filtered list of high quality variants was annotated using ANNOVAR [7]. To remove germline variants we initially filtered out common population variants from in dbSNP v38 (http://www.ncbi.nlm.nih.gov/SNP/), 1000 genomes and SNVs identitified through the above-described procedure in normal healthy thyroids. Moreover, we removed nucleotide variants located in super-duplicated regions. However, we retained those variants annotated as somatic mutations in COSMIC database. Then, we selected protein-altering point mutations (missense and nonsense mutations) and frameshift that originate from INDELs. Avsift and MA-score algorithms, implemented in ANNOVAR, were used to assess the damaging potential of the variants identified. A list of so-selected candidate nucleotide variants was analyzed using IntOGen, an integrative platform [8] that summarize somatic mutations, genes and pathway involved in tumorigenesis.

RT-PCR assay, cloning and Sanger sequencing

Reverse-Transcription Polymerase-Chain-Reaction (RT-PCR) and Sanger sequencing were used to analyze the novel candidate fusion transcripts and the novel mutations in PTC and healthy samples. cDNA synthesis and PCR amplification were performed using standard protocols that come with Superscript II Reverse Transcriptase (Invitrogen) in a 20 μl reaction according to provided protocol. PCR primers were designed to amplify 200-400 bp fragments containing the putative nucleotide variant or the gene fusion boundary, as indicated by RNA-Seq. Where multiple PCR products were detected, we cloned these amplicons into Topo Vector II plasmid (Invitrogen) according to manufacturer's instructions. PCR

products - and plasmids containing PCR amplicons - were then analyzed by direct Sanger Sequencing. Analysis of Sanger chromatograms was performed using ApE software (http://biologylabs.utah.edu/jorgensen/wayned/ape/). Refinement of chimeric transcripts' structure was performed using UCSC Blat tool. The primers used for PCR validations are reported in Supplementary Table 3.

References cited in the Supplementary Methods

1. Kim D, Pertea G, Trapnell C, Pimentel H, Kelley R, Salzberg SL. TopHat2: accurate alignment of transcriptomes in the presence of insertions, deletions and gene fusions. Genome Biol. 2013;14:R36.
2. Anders S, Pyl PT, Huber W. HTSeq-a Python framework to work with high-throughput sequencing data. Bioinformatics. 2014;pii:btu638
3. Huang DW, Sherman BT, Lempicki RA. Systematic and integrative analysis of large gene lists using DAVID Bioinformatics Resources. Nature Protoc. 2009;4(1):44-57.
4. Kim D, Salzberg SL. TopHat-Fusion: an algorithm for discovery of novel fusion transcripts. Genome Biol. 2011;12:R72.
5. Iyer MK, Chinnaiyan AM, Maher CA. ChimeraScan: a tool for identifying chimeric transcription in sequencing data. Bioinformatics. 2011;27:2903-4.
6. Van der Auwera GA, Carneiro MO, Hartl C, Poplin R, Del Angel G, Levy-Moonshine A, et al. From FastQ data to high confidence variant calls: the Genome Analysis Toolkit best practices pipeline. Curr Protoc Bioinformatics. 2013;1111.10.1-11.10.33
7. Wang K, Li M, Hakonarson H. ANNOVAR: functional annotation of genetic variants from high-throughput sequencing data. Nucleic Acids Res. 2010;38:e164
8. Gonzalez-Perez A, Perez-Llamas C, Deu-Pons J, Tamborero D, Schroeder MP, Jene-Sanz A, et al. IntOGen-mutations identifies cancer drivers across tumor types. Nat Methods. 2013;10:1081-2

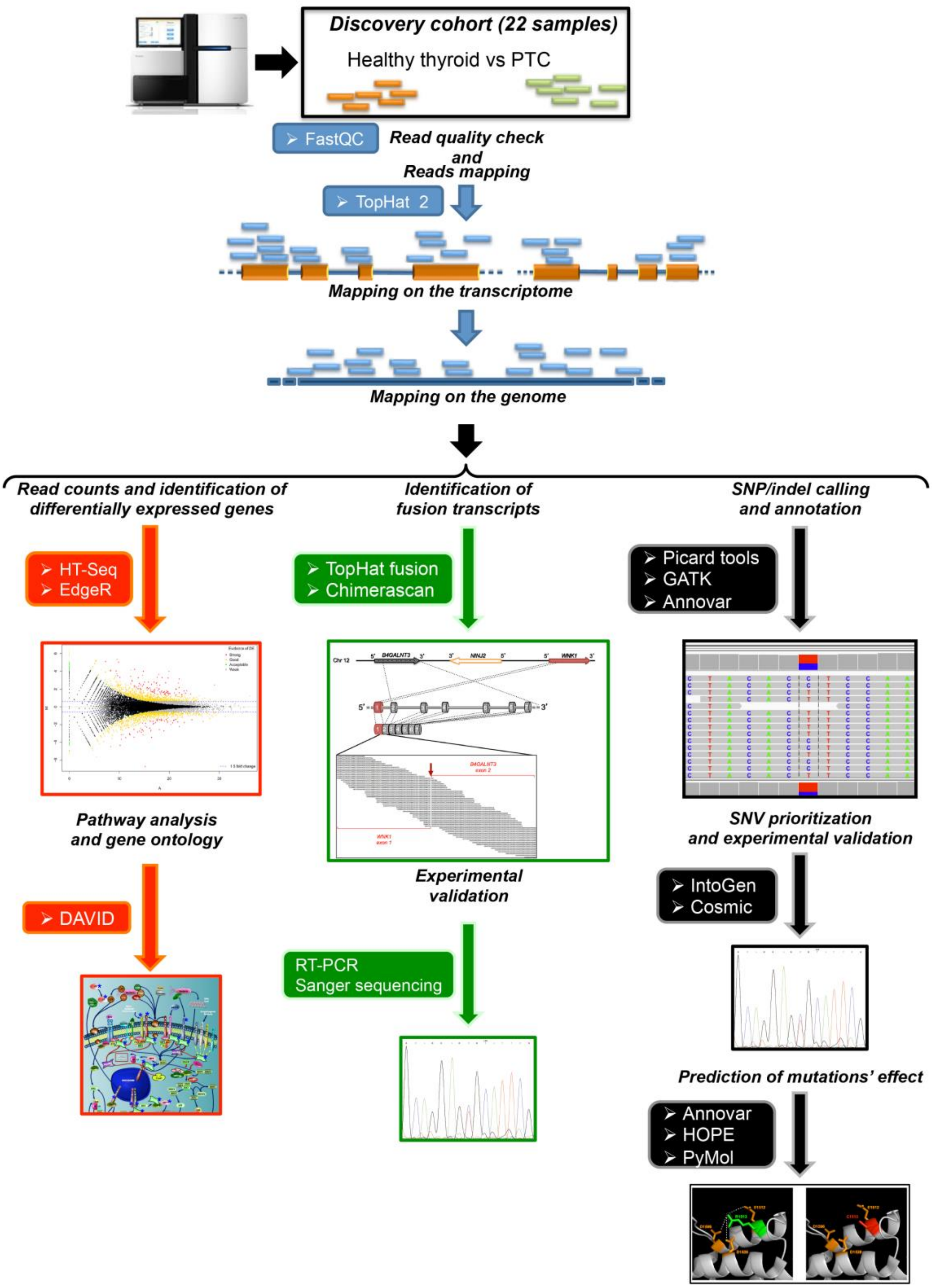

Supplementary Figure 1: Pipeline of RNA-sequencing data analysis. The first step of the analysis, consisting in reads' quality check, alignment to the known transcriptome and reference genome are shown in light blue. Reads count and analysis of differentially expressed genes are highlighted in red. Gene fusion analysis is shown in green. The variant calling procedure to identify and select point mutations and INDELs is depicted in black. The software and the databases used in this work are also indicated.

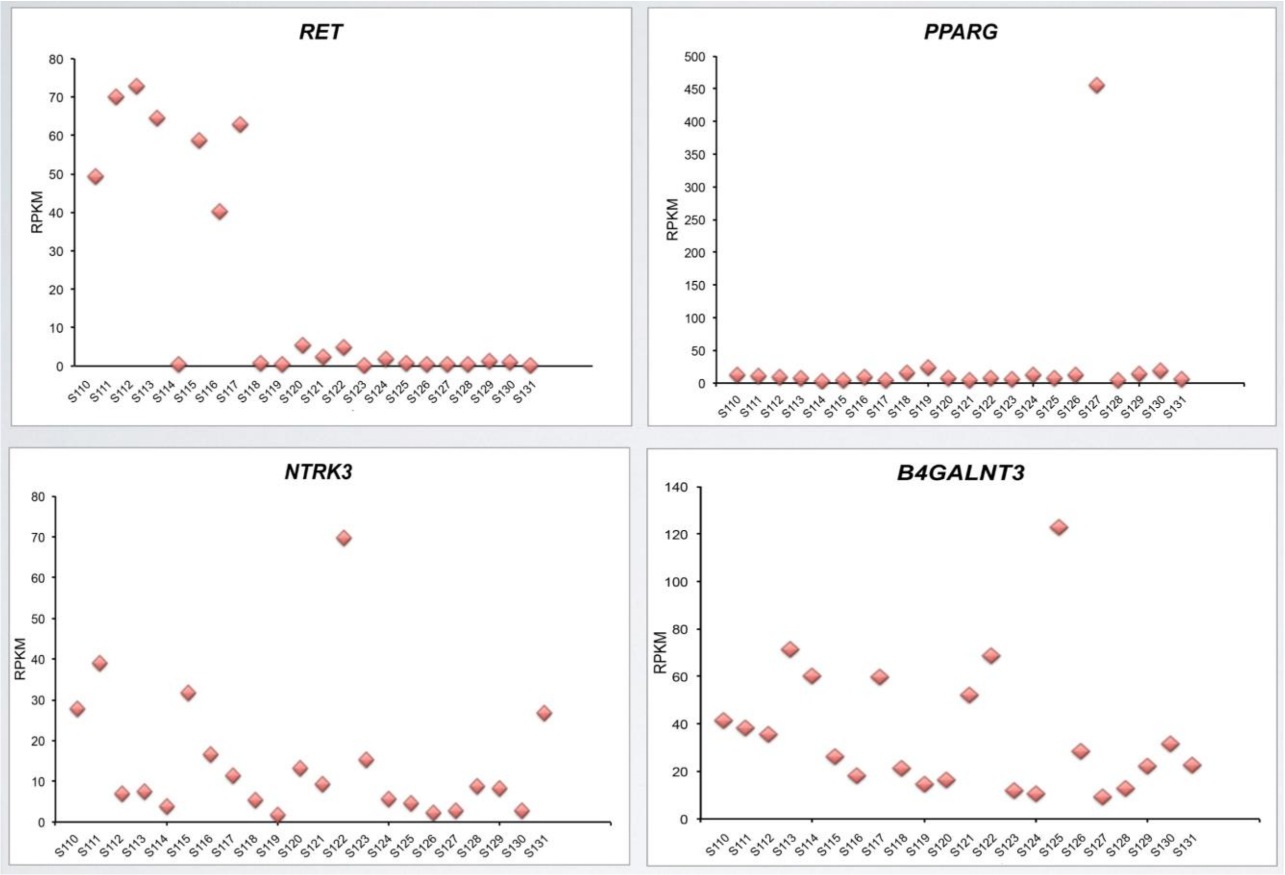

Supplementary Figure 2: Over-expression of gene fusion partners in PTC samples. Scatter chart with RPKM values (y axis) of RET, PPARG, NTRK3, and B4GALNT3 genes in PTC samples (y axis). All these genes, partners of the gene fusion, are significantly activated in PTC samples carrying the fusion.

Supplementary Table1: Summary of data generated from Paired-End RNA-Sequencing

Sample id	N° of reads	N° of fragments	Uniquely mapped reads	% of uniquely mapped reads
**S110**	107.168.970	53.584.485	97.499.398	91.0
**S111**	129.387.234	64.693.617	116.552.563	90.1
**S112**	123.056.226	61.528.113	112.010.077	91.0
**S113**	111.741.882	55.870.941	101.768.707	91.0
**S114**	112.356.482	56.178.241	100.998.996	89.9
**S115**	132.342.772	66.171.386	120.456.084	91.0
**S116**	109.612.414	54.806.207	99.716.914	91.0
**S117**	118.011.794	59.005.897	104.854.674	88.8
**S118**	151.418.292	75.709.146	137.861.418	82.0
**S119**	168.167.376	84.083.688	149.849.046	89.1
**S120**	134.011.794	68709146	121.027.091	90.3
**S121**	51.495.606	25.747.803	46.953.037	91,2
**S122**	64.766.780	32.383.390	59.983.274	92,6
**S123**	44.472.128	22.236.064	41.329.664	92,9
**S124**	64.547.635	32.273.818	59.009.673	91,4
**S125**	53.749.964	26.874.982	49.701.258	92,5
**S126**	58.995.744	29.497.872	54.237.927	91,9
**S127**	64.557.056	32.278.528	59.909.442	92,8
**S128**	70.098.721	35.049.361	65.036.396	92,8
**S129**	54.383.090	27.191.545	49.965.488	91,9
**S130**	97.514.456	48.757.228	89.589.008	91,9
**S131**	135.610.671	67.805.335	125.073.775	92,2

Supplementary Table 2: Confirmed mutations in PTC samples.

Gene	Genomic position	Nucleotide change	Amino acid change	Frequency	*Status*	Other cancer
CBL*	11:119155974	C1639T	P547S	0.02/50	heterozygous	-
NOTCH1*	9:139402738	G3271A	G1091S	0.021/47	homozygous	-
PIK3R4*	3:130447468	A1646G	E549D	0.055/18	heterozygous	-
SMARCA4*	19:11169467	C4447T	R1483C	0.055/18	heterozygous	-
MET#	7:116339642	G561T	E187D	0.02/49	heterozygous	Hematopoietic, lymphoid, endometrium, lung
DICER1#	14:95557629	A5438G	E1813G	0.021/47	heterozygous	Brain, uterus
VHL#	3:10183605	C74T	P25L	0.055/18	heterozygous	Kidney

***** completely new mutations;

known mutations never described in PTC

Supplementary Table 3: Oligolucleotides used for validations of fusion transcripts and mutations in known and new driver genes.

Gene	Forward primer	Reverse primer
BRAF	CATAATGCTTGCTCTGATAGG	TCTAGTAACTCAGCAGCATCT
CBL	GTGGGTTTTTACTGATTTGCTT	AGGGCAATGAAAATGGAAGTG
DICER1	CTGAGGAGGATGAAGAGAAAG	CTAAAGGGAGCCAACAATACC
HRAS	CCGGAAGCAGGTGGTCATTG	GCCAGCCTCACGGGGTTCA
MET	TCCCCACAATCATACTGCTG	CCATCTTTCGTTTCCTTTAGC
NOTCH1	GCAGCCTGGGTTGGAGTAGG	TCAACACCTGCGGGGGATGG
SMARCA4	CGGTGTTGGGTGTTCCTTCA	TGGGATTACAGGCACGAACC
VHL	CTGGATCGCGGAGGGAATG	AGGCGGCAGCGTTGGGTAG
B4GALNT3	-	CTCTGGGGGATGGTAGAACTGG
WNK1	CGGTCTACAAAGGTCTGGAC	GCGGTGAATGATAGGTGGAG
WNK1-B4GALNT3	CGGTCTACAAAGGTCTGGAC	GGCGGTCCACTCCTTTCCA
PIK3R4	CTATCTGTATGGGGAAAAATTG	AGATTGCATGGAAGTATTTGAG
